# Phosphorus uptake and rhizosphere properties of alfalfa in response to phosphorus fertilizer types in sandy soil and saline-alkali soil

**DOI:** 10.3389/fpls.2024.1377626

**Published:** 2024-05-10

**Authors:** Tianchan Zhang, Weifan Wan, Zhi Sun, Haigang Li

**Affiliations:** Inner Mongolia Key Laboratory of Soil Quality and Nutrient Resources, Key Laboratory of Agricultural Ecological Security and Green Development at Universities of Inner Mongolia Autonomous Region, College of Grassland, Resources and Environment, Inner Mongolia Agricultural University, Hohhot, Inner Mongolia, China

**Keywords:** alfalfa, phosphorus fertilizer, sandy soil, saline-alkali soil, soil type, rhizosphere process

## Abstract

**Introduction:**

Phosphorus (P) fertilizer is critical to maintain a high yield and quality of alfalfa (Medicago sativa L.). There are several fertilizer types and soil types in China, and the application of a single type of P fertilizer may not be suitable for present-day alfalfa production.

**Methods:**

In order to select the optimal combination of alfalfa and soil type and fertilizer type for improving P utilization efficiency. We conducted a greenhouse pot experiment, calcium superphosphate (SSP), diammonium phosphate (DAP), ammonium polyphosphate (APP), potassium dihydrogen phosphate (KP), and no-fertilizer control treatments were applied to alfalfa in sandy and saline-alkali soils. The response of alfalfa root morphology and rhizosphere processes to different P fertilizers was investigated.

**Results and discussion:**

The results showed that shoot biomass of alfalfa was slightly higher in sandy soil than in saline–alkali soil. Shoot biomass of alfalfa increased by 223%-354% in sandy soil under P treatments compared with the control, and total root length increased significantly by 74% and 53% in DAP and SSP treatments, respectively. In saline–alkali soil, alfalfa shoot biomass was significantly increased by 229% and 275% in KP and DAP treatments, and total root length was increased by 109% only in DAP treatment. Net P uptake of alfalfa in DAP treatment was the highest in both soils, which were 0.73 and 0.54 mg plant^-1^, respectively. Alfalfa shoot P concentration was significantly positively correlated with shoot and root biomass (*P* < 0.05, 0.01 or 0.001) whereas negatively correlated with acid phosphatase concentration (*P* < 0.05). Improvement of plant growth and P uptake induced by P fertilizer application was greater in sandy soil than in saline–alkali soil. DAP and KP was the most efficient P fertilizers in both sandy soil and saline–alkali soil.

## 1 Introduction

Phosphorus (P) is an essential element for plant growth (Lambers et al., 2008). Not only is it a component of nucleic acids, proteins, and other organic compounds in plants but it also participates in photosynthesis, enzyme activity regulation, carbon cycle and other nutrient cycles, and energy metabolism; regulates physiological and biochemical processes; and plays an irreplaceable role in plant growth and development, yield, and quality determination (Vance, 2001; Vance et al., 2003; Plaxton and Tran, 2011). Being non-renewable, the mineral resources of P in the earth’s crust will be sufficient only for 50 to 200 years (Baker et al., 2015). The P forms that can be directly absorbed by plants are mainly H_2_PO_4_^-^ and HPO_4_^2-^ in soil solution. Organic P in soil needs to be converted to these inorganic P forms before it can be absorbed by plant roots. The distribution of P in soil is characterized by surface accumulation and poor mobility. Soluble P readily forms insoluble compounds with metal ions, such as AlPO_4_, FePO_4_, Fe_3_(PO_4_)_2_, and Ca_3_(PO_4_)_2_, or phosphate ions are adsorbed on the surface of soil minerals, owing to which the use efficiency of P fertilizers in wheat (*Triticum aestivum* L.) and white lupin (*Lupinus albus* L.) during the first growing season is only 10%–20% (Bünemann et al., 2006; Cornish, 2009). Thus, P availability is a limiting factor in crop production in many developing countries, especially P reserve-poor countries (Richardson et al., 2011). Because of the low P use efficiency, farmers typically need to apply more P fertilizer than the crops require to sustain yield. This practice not only wastes resources but also increases the risk of soil P loss through leaching and soil erosion, which poses a threat to ecological environments.

For adaptation to P-deficient environments, plants have evolved various strategies to improve P utilization efficiency internally and increase P absorption externally (Vance et al., 2003; Baker et al., 2015). The strategies to improve P utilization efficiency include modifications in root morphology, regulation of rhizosphere pH, carboxylate exudation, acid phosphatase (APase) secretion, association with arbuscular mycorrhizal fungi, and upregulation of high-affinity P transporters (Chen et al., 2007; López-Arredondo et al., 2014). For example, white lupin forms cluster roots to cope with P deficiency stress (Lamont, 2003). P-deficient plants tend to increase the root/shoot ratio and root length by allocating more photosynthetic products to roots (Liao et al., 2001; Lynch and Brown, 2001). In low P alkaline soils, alfalfa (*Medicago sativa* L.) enhances rhizosphere acidification and carboxylate exudation (Fan et al., 2015; He et al., 2017; Wang et al., 2020). APase facilitates the hydrolysis of soil organic P, and it has been reported that its activity in the rhizosphere of P-deficient plants is higher than that in P-sufficient plants (George et al., 2008; Ma et al., 2021).

Alfalfa is a perennial leguminous forage crop with high nutritional value, as it is rich in proteins, vitamins, and minerals. It is also a plant with a high P demand, and each ton of alfalfa hay contains approximately 2.7 kg P (Undersander et al., 2021). In China, alfalfa often grows in marginal land with poor soil fertility, especially regarding P (Wan et al., 2022). Thus, P fertilizer application is necessary to maintain a high yield and quality of alfalfa. Many types of P fertilizers are available on the market, such as single superphosphate (SSP), monoammonium phosphate (MAP), diammonium phosphate (DAP), and ammonium polyphosphate (APP) (Li et al., 2016). Azeem et al. (2018) found DAP delayed maize maturity compared with triple superphosphate (TSP) and SSP in a clay loam soil (pH 6.5-7.8). Previous studies shows that SSP has a similar efficiency with DAP to improve in maize yield although the former’s price is generally lower than the latter (Amanullah et al., 2009). In a calcareous soil, alfalfa growth did not show a significant difference between granular MAP and liquid APP application (Ottman et al., 2006). By far, few studies focus on P fertilizer application of alfalfa in China. There are many of P fertilizers types in the market, and it is difficult for farmers to choose a suitable fertilizer for their alfalfa production in absence of a reliable guide because research remains limited to date. Therefore, in the present study, we tested (1) the effect of four P fertilizer types on improvement in alfalfa yield and (2) the response of rhizosphere processes of alfalfa grown in poor soils collected from marginal land to different P fertilizer types.

## 2 Materials and methods

### 2.1 Plants, soil, and experimental design

Two types of soil samples were collected respectively from Urad Middle Banner, Bayannur City (41°34′N, 108°31′E; saline–alkali soil) and Dalad Banner Ordos (40°20′N, 110°45′E; for sandy soil) in Inner Mongolia Autonomous Region, China. Air-dried soil samples were sieved through a 2-mm sieve. Soil properties in saline–alkali soil were: total N 0.39 g/kg, Olsen-P 3.26 mg/kg, available K 88.24 mg/kg, pH 8.75 (1:5, soil:water). Soil properties in sandy soil were: total N 0.44 g/kg, Olsen-P 7.12 mg/kg, available K 92.58 mg/kg, pH 7.71 (1:5, soil:water).

The cultivar of ‘Zhongmu No.1’ was chose in this study because it is widely cultivated in northern China as a salinity cold and drought tolerance cultivar (Long et al., 2011; Liu et al., 2022). To ensure an adequate supply of other nutrients for alfalfa growth, soil samples were supplemented with basic nutrients in the following quantities (mg kg^−1^ soil): N, 200 (when N added to P fertilizer is less than 200 mg kg^−1^, make up the amount in the form of Ca(NO_3_)_2_·4H_2_O); K, 200 (when K added to P fertilizer is less than 200 mg kg^−1^, make up the amount in the form of K_2_SO_4_); CaCl_2_, 125.67; MgSO_4_·7H_2_O, 43.34; EDTA-FeNa, 5.80; MnSO_4_·4H_2_O, 6.67; ZnSO_4_·7H_2_O, 10.00; CuSO_4_·5H_2_O, 2.00; H_3_BO_3_, 0.67; and (NH_4_)_6_Mo_7_O_24_·4H_2_O, 0.26. The following phosphate fertilizers were tested in this study: calcium superphosphate (Sinopharm Chemical Reagent Co., Ltd.), diammonium phosphate (Sinopharm Chemical Reagent Co., Ltd.), ammonium polyphosphate (Sinopharm Chemical Reagent Co., Ltd.), and potassium dihydrogen phosphate (Sinopharm Chemical Reagent Co., Ltd.).

P fertilizers were uniformly mixed with air-dried soil samples in the respective treatment groups at a rate of 60 mg P kg^−1^, which is the critical level for alfalfa growth based on our previous study. Control treatments without any P addition were established. There were four replicates (pots) per treatment group, and a completely random design was adopted. Eight germinated seeds were sowed per 1 L pot (1 kg soil per pot), and each pot was thinned to four seedlings after 1 week. Throughout the plant growth period, the soil moisture in each pot was maintained at 75% of the field capacity by watering using the weighing method. The plants were harvested at 37 days after sowing.

### 2.2 Collection and determination of root exudates

The plants were gently lifted from the soil and shaken lightly to remove bulk soil from the root systems. The roots were inserted into a 50 mL vial containing 0.2 mM CaCl_2_ solution and shaken gently to collect as much rhizosphere soil as possible. After removing the roots from the vial, two 0.5 mL aliquots of soil suspension were transferred to 2 mL centrifuge tubes to determine the activity of APase. APase activity was measured on the same day. Ten mL of soil suspension was transferred to another centrifuge tube for the determination of carboxylates in the rhizosphere soil. Two drops of 0.01 g L^−1^ microbial inhibitor (MICROPUR MP1, Katadyn Products Inc. Minneapolis, MN 55429 USA, Germany) and 2 drops of concentrated phosphoric acid were added to inhibit microbial decomposition of the sample. Then, the samples were stored at −20°C until analysis using high-performance liquid chromatography (Alvey et al., 2001).

Determination of APase activity in rhizosphere soil: 0.4 mL of sodium acetate buffer and 0.1 mL of p-nitrophenyl phosphate (p-NPP) were added to the sample; after incubation at 25°C for 30 min, 0.5 mL of 0.5 M NaOH was added to terminate the reaction. APase activity was measured using spectrophotometry at 405 nm (Tabatabai and Bremner, 1969).

Determination of carboxylates in rhizosphere soil: Thawed samples were filtered through a 0.22 µm filter membrane, and high-performance liquid chromatography was used for the determination of carboxylates in rhizosphere soil. The chromatographic separation was conducted on a 250 × 4.6 mm reversed-phase column (Alltima C18, 5 µm; Alltech Associates, Inc., Deerfield, IL, USA). The mobile phase was 25 mmol L^−1^ KH_2_PO_4_ (pH 2.25) with a flow rate of 1 mL min^−1^ at 37°C. Detection of carboxylates was performed at 214 nm (Shen et al., 2005).

Δ Soil pH calculated as bulk soil pH minus rhizosphere soil pH.

### 2.3 Determination of root morphological parameters

The roots were scanned with an EPSON scanner (Epson Perfection V800 Photo/V800 Pro, Model EU-35, Japan), and the images were analyzed using the WinRHIZO image analysis system (Regent Instrument, Quebec, Canada) to determine root parameters such as total root length and root diameter.

Specific root length is the ratio of total root length to root dry weight.

### 2.4 Determination of alfalfa biomass and phosphorus concentration and soil Olsen-P

Shoots and roots were washed with deionized water. Shoots and roots were dried at 70°C for 3 days to constant weight. The plant biomass indicators were determined by weighing the samples on a balance. The samples were ground into powder to perform plant P analysis.

Samples were digested with concentrated H_2_SO_4_ and H_2_O_2._ The total P concentration in the digest was measured total P using the molybdate blue colorimetric method (Murphy and Riley, 1962).

The air-dried soil samples were extracted with 0.5 M NaHCO_3_ (pH = 8.5) and analyzed using the molybdo-vanado-phosphatase method (Westerman, 1990).

The net P uptake per plant was calculated as follows: *Net P uptake* = (*Shoot P concentration* × *Shoot biomass*) + (*Root P concentration* × *Root biomass*)

The P absorption efficiency per plant was calculated as follows: *P absorption efficiency* = *Net P uptake/Total root length* (Lyu et al., 2016).

### 2.5 Statistical analysis

All data were calculated and organized using Excel 2016 (Microsoft, USA). Plant and rhizosphere soil indicator data were analyzed using the one-way analysis of variance (ANOVA) model in SAS statistical software (SAS9.2, SAS Institute Inc., Cary, NC, USA). Significant differences among means were determined using LSD test at the P <0.05 probability level. The SigmaPlot statistical software (SigmaPlot 12.5, Systat Software Inc., San Jose, CA, USA) was adopted to create the bar charts. The Originpro 2021 (Microsoft, USA) was adopted to create the matrix of Spearman’s correlation coefficients. We used Amos Graphics software (IBM Corp., Armonk, NY, USA) to construct the structural equation models and Microsoft Office Visio 2007 (Microsoft, USA) to optimize the graphs.

## 3 Results

### 3.1 Plant growth and P uptake

**Figure 1:**
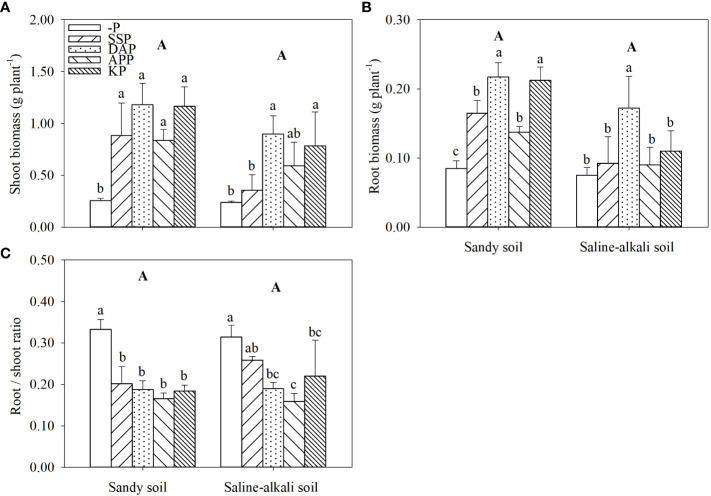
Shoot **(A)** and root **(B)** biomass and root/shoot ratio **(C)** of alfalfa grown under different fertilizer treatments in sandy soil and saline–alkali soil. Each value is the mean (± standard deviation [SD]) of four replicates. Different capital letters indicate significant differences among soil types, and different lowercase letters indicate significant differences among P fertilizer types (*P* ≤ 0.05). -P, no phosphate fertilizer treatment; SSP, calcium superphosphate treatment; KP, KH_2_PO_4_ treatment; DAP, diammonium phosphate treatment; APP, ammonium polyphosphate treatment. The abbreviations in all subsequent images are the same and therefore have not been defined in each legend below.

Both shoot and root biomass of alfalfa showed no significant difference between soil types (**Figures 1A, B**; **Supplementary Figure S1**). In sandy soil, the shoot biomass of alfalfa in the APP, SSP, KP, and DAP treatments was 223%, 238%, 350%, and 354% higher than that in the control, respectively (**Figure 1A**). The shoot biomass reached up to 1.17 g plant^−1^ in the KP treatment and 1.18 g plant^−1^ in DAP treatment. In contrast, in saline–alkali soil, the APP and SSP treatments did not improve shoot growth. However, the shoot biomass in the KP and DAP treatments in saline–alkali soil (0.79 g and 0.90 g plant^−1^, respectively) was significantly higher (by 229% and 275%, respectively) than that in the control (**Figure 1A**). In sandy soil, the root biomass in the DAP, KP, SSP and APP treatments was significantly higher (by 144%, 133%, 89%, and 56%, respectively) than that in the control (**Figure 1B**). However, in saline–alkali soil, the root biomass in the SSP, KP, and APP treatments did not show significant changes. The root biomass in the DAP treatment (0.17 g plant^−1^) was the highest and was significantly higher (by 113%) than that in the control (**Figure 1B**).

The root/shoot ratio of alfalfa was not significantly different between the two studied soil types (**Figure 1C**). In sandy soil, the root/shoot ratio in the control was significantly higher than that in P application treatments, and there was no significant difference among the SSP, DAP, APP and KP treatments. In saline–alkali soil, the root/shoot ratio of alfalfa in SSP treatment did not significantly differ from that in the control. Moreover, the root/shoot ratio in the KP, DAP, and APP treatments was significantly lower than that in the control treatment (**Figure 1C**).

**Figure 2:**
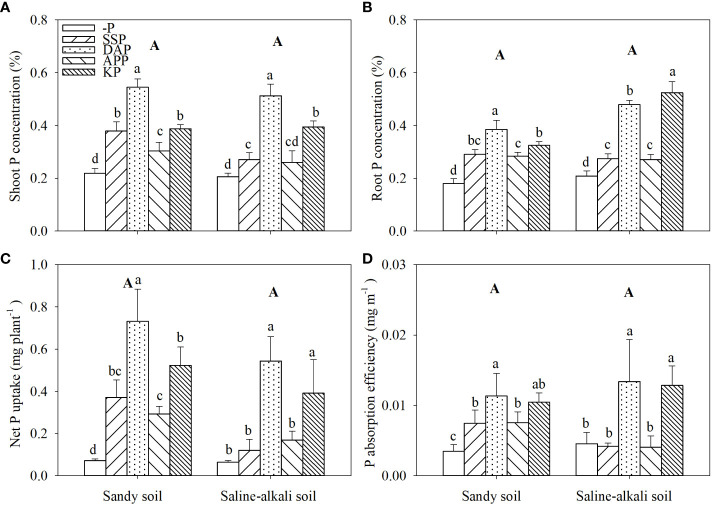
Shoot **(A)** and root **(B)** P concentration, net P uptake **(C)**, and P absorption efficiency **(D)** of alfalfa grown under different fertilizer treatments in sandy soil and saline–alkali soil. Each value is the mean (± standard deviation [SD]) of four replicates. Different capital letters indicate significant differences among soils, and different lowercase letters indicate significant differences among P fertilizer types (*P* ≤ 0.05).

No differences were observed between soil types in both shoot and root P concentrations (**Figures 2A, B**). In sandy soil, all P fertilizer applications significantly increased the shoot P concentration of alfalfa; the DAP treatment showed the highest shoot P concentration (0.55%; **Figure 2A**). The shoot P concentration in the APP, SSP, KP, and DAP treatments was 36%, 73%, 77%, and 150% higher than that in the control, respectively. In saline–alkali soil, the shoot P concentration in the SSP, APP, and KP treatments was 0.27%, 0.26%, and 0.39%, respectively, with the DAP treatment showing the highest shoot P concentration (0.51%; **Figure 2A**). In sandy soil, the root P concentration in the DAP treatment was also significantly higher than that in other treatments. The root P concentration in the DAP treatment was 0.39%, which was 132% higher than that in the control. There was no significant difference in root P concentration among SSP, APP and KP application in sandy soil, while KP application significantly increased root P concentration compared with APP (**Figure 2B**). In saline–alkali soil, the application of P fertilizers significantly increased the root P concentration of alfalfa (**Figure 2B**). The root P concentration in the SSP, APP, DAP, and KP treatments was 29%, 29%, 129%, and 148% higher than that in the control, respectively. The root P concentration in the DAP treatment reached 0.48% in saline–alkali soil, significantly higher than that in sandy soil (**Figure 2B**).

There was no difference in the net P uptake of alfalfa among soil types (**Figure 2C**). In sandy soil, the net P uptake of alfalfa in all P fertilizer treatments was higher than that in the control. The net P uptake of alfalfa was the highest at 0.73 mg plant^−1^ in the DAP treatment. In contrast, in saline–alkali soil, the application of SSP and APP did not increase the net P uptake of alfalfa. The highest net P uptake was observed in the DAP treatment (0.54 mg plant^−1^; **Figure 2C**).

In sandy soil, the root P absorption efficiency in the DAP, KP, APP, and SSP treatments was 223%, 197%, 114%, and 114% higher than that in the control, respectively (**Figure 2D**). In saline–alkali soil, the root P absorption efficiency in the DAP and KP treatments was 191% and 180% higher than that in the control, respectively. The SSP and APP treatments in saline–alkali soil did not significantly affect root P absorption efficiency (**Figure 2D**).

### 3.2 Soil Olsen-P

**Figure 3:**
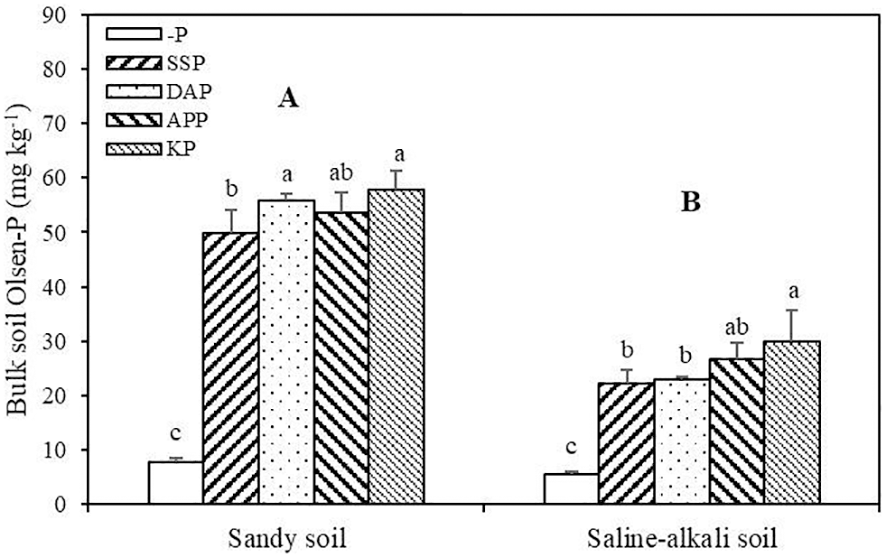
Bulk soil Olsen-P of alfalfa under different treatments in sandy soil and saline–alkali soil. Each value is the mean [± standard deviation (SD)] of four replicates. Different capital letters indicate significant differences among soils (P ≤ 0.05), and different lowercase letters indicate significant differences among P fertilizer types (*P* ≤ 0.05).

Soil Olsen-P in sandy soil was significantly higher than that in saline–alkali soil (**Figure 3**). In sandy soil, SSP, KP, DAP, and APP application significantly increased soil Olsen-P. Among P fertilizer application treatments, the KP and SSP treatments showed the highest (57.81 mg kg^−1^) and lowest soil Olsen-P (49.81 mg kg^−1^), respectively. There was no significant difference in soil Olsen-P between the DAP and APP treatments. In saline–alkali soil, the SSP, KP, DAP, and APP treatments showed significantly higher soil Olsen-P than that in the control, and the KP and SSP treatments showed the highest (30.01 mg kg^−1^) and lowest values (22.22 mg kg^−1^), respectively (**Figure 3**).

### 3.3 Root morphology

**Figure 4:**
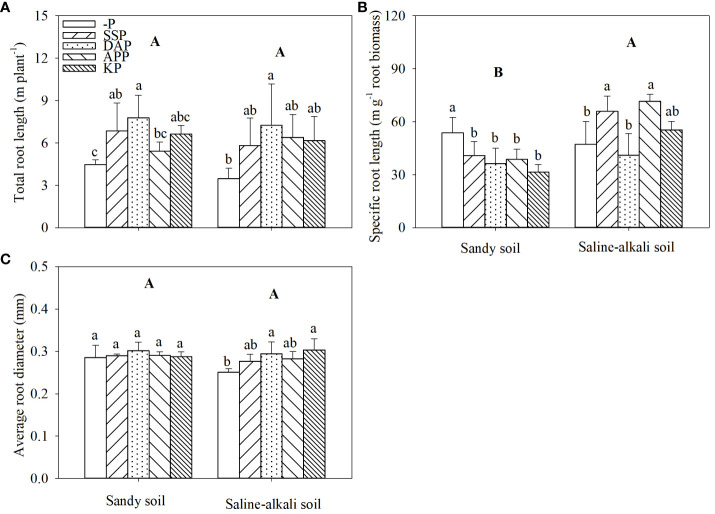
Total root length **(A)** specific root length **(B)** and average root diameter **(C)** of alfalfa under different treatments in sandy soil and saline–alkali soil. Each value is the mean [± standard deviation (SD)] of four replicates. Different capital letters indicate significant differences among soil types, and different lowercase letters indicate significant differences among P fertilizer types (*P* ≤ 0.05).

Total root length did not show a significant difference between soil types (**Figure 4A**). In sandy soil, the total root length of alfalfa was significantly higher (by 74% and 53%, respectively) in the DAP and SSP treatments than in the control (**Figure 4A**). In saline–alkali soil, only the DAP treatment showed significantly higher total root length (by 109%) than the control. SSP, APP, and KP application did not change total root length of alfalfa (**Figure 4A**). The specific root length of alfalfa in saline–alkali soil was higher than that in sandy soil (**Figure 4B**). In sandy soil, all treatments with P fertilizers showed lower specific root length than the control, and there was no significant difference in the specific root length among P fertilizer treatments. In saline–alkali soil, DAP and KP treatments did not affect the specific root length of alfalfa; however, the SSP and APP treatments showed 39% and 52% higher specific root length, respectively, than the control (**Figure 4B**). The average root diameter in sandy soil had no significant difference among the treatments. In saline-alkali soil, KP and DAP treatments were significantly higher than the control, which was 0.30 mm, and 0.29 mm, respectively (**Figure 4C**).

**Figure 5:**
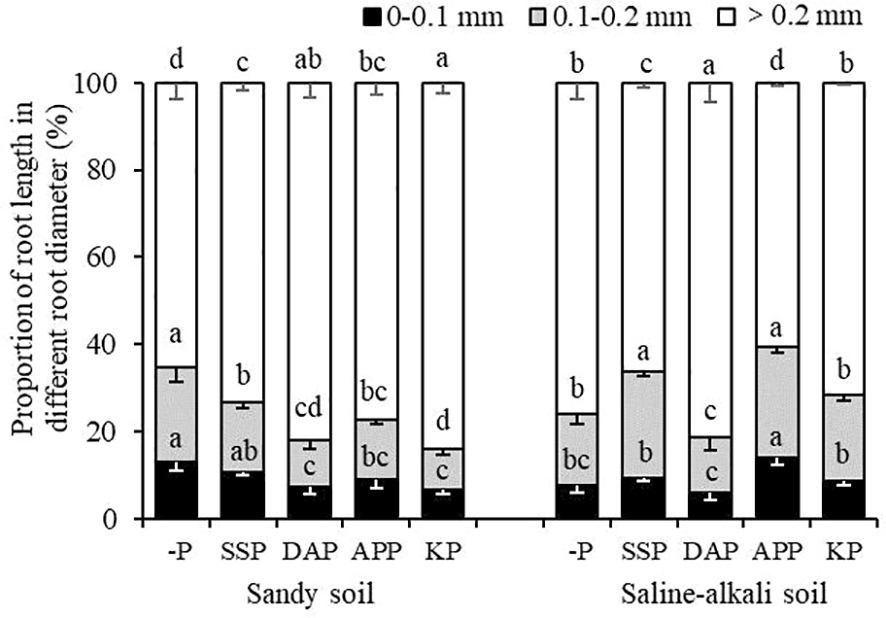
Proportion of root length in different diameter ranges to total root length of alfalfa in sandy soil and saline–alkali soil. Each value is the mean [± standard deviation (SD)] of four replicates. Different capital letters indicate significant differences among soils, and different lowercase letters indicate significant differences among P fertilizer types (*P* ≤ 0.05).

In sandy soil, SSP application did not significantly affect the proportion of root length within 0–0.1 mm, whereas DAP, APP, and KP treatments showed a significantly lower proportion of root length within 0–0.1 mm (by 44%, 31%, and 47%, respectively) than the control (**Figure 5**). However, the proportion of root length within 0.1–0.2 mm was highest in the control (22%) and lowest in the KP treatment (9%), and there were no significant differences among the SSP, DAP, and APP treatments. The proportion of root length within >0.2 mm was the highest in the KP treatment (84%) and lowest in the control (65%). In saline–alkali soil, the proportion of root length within 0–0.1 mm in APP treatment was highest (14%) and lowest in the DAP treatment (6%), and there was no significant difference between the control, SSP, and KP treatments. The proportion of root length within 0.1–0.2 mm in the APP and SSP treatments were significantly higher than the other treatments, and that in the DAP treatment was the lowest (13%). The proportion of root length within >0.2 mm was the highest in the DAP treatment (81%) and lowest in the control (8%; **Figure 5**).

### 3.4 Carboxylate concentration, acid phosphatase activity, and pH in the rhizosphere

The predominant carboxylates in the rhizosphere soil of alfalfa were malate, citrate, succinate, and tartrate (**Table 1**). The concentration of malate and tartrate were not significant different between sandy soil and saline–alkali soil, and the application of P fertilizers did not significantly affect their concentration. In the SSP treatment, citrate concentration in the rhizosphere was higher in sandy soil than in saline–alkali soil. In sandy soil, the citrate concentration in the rhizosphere did not significantly differ among P fertilizer treatments, and the highest concentration of succinate (1.45 µmol g^−1^ soil) was found in the control treatment. In saline–alkali soil, the concentration of citrate and succinate did not significantly differ among treatments (**Table 1**).

**Table 1:** Carboxylate concentration in the rhizosphere of alfalfa grown under different fertilizer treatments in sandy soil and saline–alkali soil.

	Rhizosphere carboxylate (μmol per g soil)							
	Malate		Citrate		Succinate		Tartrate	
	Sandy soil	Saline-alkali soil	Sandy soil	Saline-alkali soil	Sandy soil	Saline-alkali soil	Sandy soil	Saline-alkali soil
-P	0.70 ± 0.20 Aa	1.01 ± 0.36 Aa	1.79 ± 0.60 Aa	0.36 ± 0.10 Aa	1.45 ± 0.44 Aa	0.14 ± 0.24 Ba	0.31 ± 0.13 Aa	0.36 ± 0.05 Aa
SSP	0.71 ± 0.23 Aa	1.00 ± 0.31 Aa	1.08 ± 0.39 Aa	0.30 ± 0.18 Ba	0.63 ± 0.38 Abc	0.39 ± 0.39 Aa	0.22 ± 0.16 Aa	0.27 ± 0.07 Aa
DAP	0.51 ± 0.11 Aa	0.73 ± 0.29 Aa	0.74 ± 0.32 Aa	0.63 ± 0.26 Aa	0.62 ± 0.20 Abc	0.49 ± 0.50 Aa	0.21 ± 0.30 Aa	0.51 ± 0.42 Aa
APP	0.72 ± 0.35 Aa	0.62 ± 0.62 Aa	0.70 ± 0.12 Aa	0.56 ± 0.28 Aa	0.32 ± 0.19 Ac	0.25 ± 0.26 Aa	0.20 ± 0.13 Aa	0.40 ± 0.54 Aa
KP	0.99 ± 0.33 Aa	0.78 ± 0.28 Aa	1.28 ± 0.55 Aa	0.62 ± 0.33 Aa	1.11 ± 0.15 Aab	0.52 ± 0.42 Aa	0.28 ± 0.20 Aa	0.23 ± 0.16 Aa

Each value is the mean (± standard deviation [SD]) of four replicates. Different capital letters indicate significant differences among soil types, and different lowercase letters indicate significant differences among P fertilizer types (*P* ≤ 0.05).

**Figure 6:**
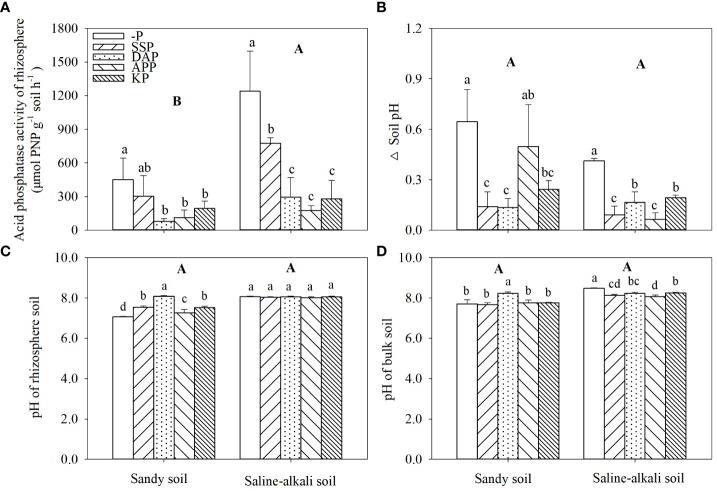
Changes in acid phosphatase activity in rhizosphere **(A)** and Δ soil pH **(B)** (calculated as bulk soil pH minus rhizosphere soil pH) pH of rhizosphere **(C)** and bulk soil **(D)** under different fertilizer treatments in sandy soil and saline–alkali soil. Each value is the mean (± standard deviation [SD]) of four replicates. Different capital letters indicate significant differences among soils, and different lowercase letters indicate significant differences among P fertilizer types (*P* ≤ 0.05).

APase activity in saline–alkali soil was higher than that in sandy soil (**Figure 6A**). In sandy soil, APase activity in the rhizosphere was 450 and 302 µmol PNP h^−1^ g^−1^ soil in the control and SSP treatment, respectively, which was significantly higher than that in other treatments. APase activity in the DAP treatment was the lowest, which was 76 µmol PNP h^−1^ g^−1^ soil. In saline–alkali soil, APase activity in the control group was 1239 µmol PNP h^−1^ g^−1^ soil, which was significantly higher than that in the P fertilizer treatments. Moreover, APase activity in the SSP treatment was significantly higher than that in the KP, DAP, and APP treatments (**Figure 6A**).

Alfalfa rhizosphere acidification occurred in sandy soil and saline–alkali soil, but there was no significant difference between soil types (**Figure 6B**). In sandy and saline–alkali soils, the control without P application decreased the rhizosphere pH by 0.65 and 0.41. In sandy soil, both the rhizosphere (8.09) and bulk soil (8.22) pH in the DAP treatment were significantly higher than the other treatments (**Figures 6C, D**). In saline–alkali soil, there was no change in rhizosphere soil pH among treatments, while P fertilizer application significantly decreased bulk soil pH compared to the control.

### 3.5 Linkages of alfalfa growth and P uptake with soil Olsen-P, root morphology, and rhizosphere processes

**Figure 7:**
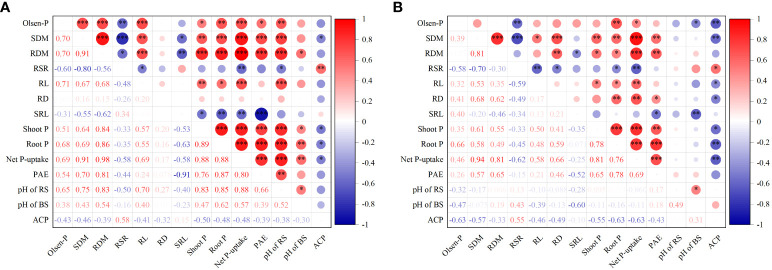
Spearman’s correlations of alfalfa growth and P uptake with soil Olsen-P, root morphology, and rhizosphere processes in sandy **(A)** and saline–alkali soil **(B)**. Olsen-P, soil Olsen-P; SDM, shoot dry mass; RDM, root dry mass; RSR, root/shoot ratio; RL, root length; RD, root diameter; SRL, specific root length; Shoot P, shoot P concentration; Root P, root P concentration; PAE, P absorption efficiency; pH of RS, pH of rhizosphere soil; pH of BS, pH of bulk soil; and ACP, acid phosphatase activity. *P < 0.05, **P < 0.01, and ***P <0.001.

We analyzed the correlations alfalfa growth and P uptake with soil Olsen-P, root morphology and rhizosphere processes in sandy and saline–alkali soils (**Figure 7**). In sandy soil, shoot biomass was significantly positively correlated with soil Olsen-P, root length, shoot P concentration, root P concentration, root P absorption efficiency, and (*P* < 0.01 or 0.001), and negatively correlated with specific root length and APaseactivity in the rhizosphere (*P* < 0.05; **Figure 7A**). Net P uptake of alfalfa showed a significant positive correlation with soil Olsen-P, root length, root P absorption efficiency, and pH of rhizosphere and bulk soil (*P* < 0.01 or 0.001), while it was negatively correlated with specific root length and APase activity in the rhizosphere (*P* < 0.05). Soil Olsen-P in sandy soil was positively correlated with root length, shoot and root P concentration, root P absorption efficiency, and rhizosphere soil pH of alfalfa (*P* < 0.05 or 0.01 or 0.001), and significantly negatively correlated with root/shoot ratio (*P* < 0.01). In saline–alkali soil, shoot biomass was positively correlated with root length, root diameter, shoot and root P concentration, and root P absorption efficiency (*P* < 0.05, 0.01 or 0.001), whereas it was significantly negatively correlated with APase activity (*P* < 0.01; **Figure 7B**). Net P uptake was positively correlated with soil Olsen-P, root length, root diameter, and root P absorption efficiency (*P* < 0.05 or 0.01 or 0.001), and was also significantly negatively correlated with APase activity (*P* < 0.01). Soil Olsen-P was significantly positively correlated with shoot P concentration of alfalfa (*P* < 0.01), and negatively correlated with root/shoot ratio, pH of bulk soil and APase activity (*P* < 0.05, 0.01 or 0.001).

**Figure 8:**
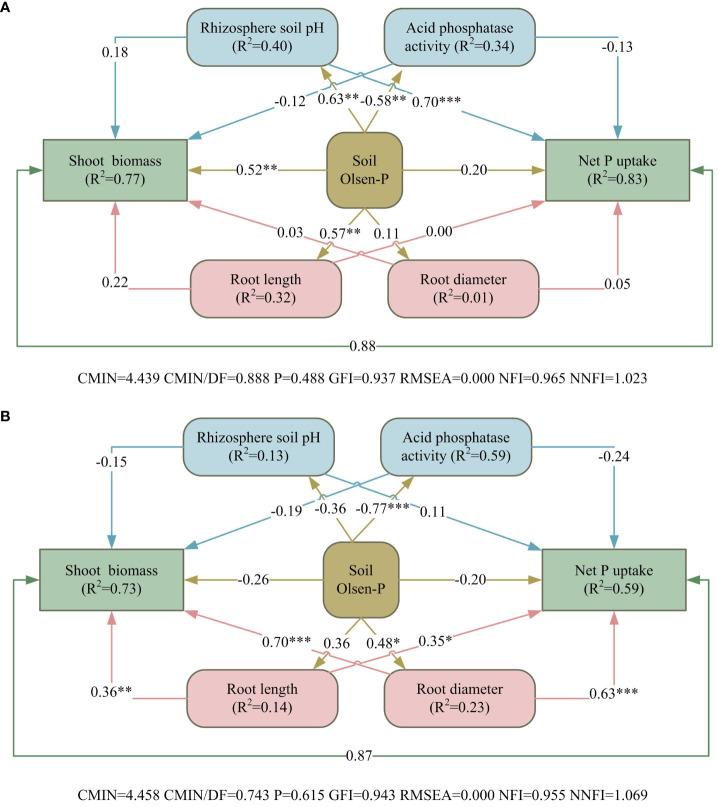
Structural equation models revealing the direct and indirect effects of rhizosphere processes, soil Olsen-P and root morphology on alfalfa growth in sandy **(A)** and saline–alkali soil **(B)**. Arrows represent hypothesized causal relationships between variables. The coefficients in black solid lines are standardized prediction coefficients for each causal path. R^2^ indicates the total variation of a dependent variable is explained by independent variables. *P < 0.05, **P < 0.01, and ***P <0.001.

Among the many predictor variables in sandy soil, pH of the rhizosphere soil, APase activity, Olsen-P, root length, and root diameter accounted for 77% of the effect on alfalfa shoot biomass, and these variables had up to 83% of the effect on net P uptake (**Figure 8A**). Soil Olsen-P had significant positive direct effects on shoot biomass, and pH of rhizosphere soil had a highly significant positive direct effects on net P uptake. In saline–alkali soil, the above predictor variables had 73% of the effect on alfalfa shoot biomass, while had only 59% of the effect on net P uptake (**Figure 8B**). Root length and root diameter had significant direct positive effects on both aboveground biomass and net phosphorus uptake of alfalfa.

## 4 Discussion

### 4.1 Biomass and P uptake

P fertilizer application in low P soil considerably improved not only hay yield, P uptake, and utilization efficiency of alfalfa but also soil available P (Sanderson and Jones, 1993; He et al., 2017, He et al., 2020). Furthermore, most P fertilizers increased alfalfa growth and P uptake, but this positive effect varied among fertilizer and soil types (**Figures 1A, B**, **2C**). This finding was also consistent with the soil Olsen-P concentration in the two soil types. Salts enhance P adsorption on the soil surface (Al-Falih, 2001; Cho-Ruk, 2003), which is often accompanied by high pH in saline–alkali soils (He et al., 1997). Soil Olsen-P did not determine alfalfa growth in saline–alkali soil. For example, despite the similar soil Olsen-P, plant growth differed between the SSP and DAP treatments (**Figures 1A, B**, **3**). In most cases, salt stress reduces P content in plant tissues (Günes et al., 1999). The shoot and root P concentration in rice decreased with the increase in salt concentration (Alam et al., 2002). Remarkably, in saline–alkali soil, acid (SSP) and neutral (APP) fertilizers caused less improvement in plant growth than the alkali (DAP) fertilizer; a likely explanation for this finding is the low P uptake efficiency than soil Olsen-P and root length (**Figures 1A, B**).

Previous studies have reported that DAP is a more efficient fertilizer among the P fertilizers used in agricultural production (Montalvo et al., 2015; He et al., 2017). In the present study, this finding was confirmed in saline–alkali soil; DAP significantly increased plant growth, P uptake, and shoot P concentration in alfalfa than SSP and APP in saline–alkali soil (**Figures 1A, B**, **2A, C**). The results suggest that DAP alleviated the salt stress of alfalfa, the mechanism of which remains unclear. NH_4_^+^ uptake in the DAP treatment did not lead to higher acidification in the rhizosphere of alfalfa compared with the other fertilizer treatments. However, this advantage of DAP disappeared in sandy soil, in which there were fewer limiting factors than saline–alkali soil. As a slow-release fertilizer, APP has been considered efficient for increasing plant P uptake because of alleviation of P adsorption by soil minerals, especially in soils with high P fixation potential (Venugopalan and Prasad, 1989; Bertrand et al., 2006). However, our results were not consistent with this finding. In saline–alkali soil, salt stress significantly inhibited the fertilizer efficiency of APP (**Figure 2D**). Soil pH in the SSP treatment was approximately 1.5 owing to the reaction of fertilizer particles with the soil (Syers et al., 2008), but it did not lower the rhizosphere pH of alfalfa, which may alleviate salt stress. Thus, it was inefficient in saline–alkali soil.

### 4.2 Root morphology

P application has been reported to increase the root length of P-deficient maize and wheat (Wen et al., 2017; Shen et al., 2018). However, our results were not consistent with this finding and showed that root length depended on soil and P fertilizer type. In both soil types, DAP caused the highest increase in root length (**Figure 4A**), which may be due to root proliferation caused by ammonium in DAP (Bloom et al., 2002). Fine roots with a larger surface area facilitates P adsorption although its turnover rate is high, which leads to a large carbon cost (Wen et al., 2019). P-deficient plants tend to increase the specific root length and product a higher number of thin roots to increase the adsorption surface (Løes and Gahoonia, 2004; Rabbi et al., 2017; Wang et al., 2018). In sandy soil, the specific root length increased with P fertilizer application (**Figure 4B**), which is consistent with previous findings (Wen et al., 2019). However, in saline–alkali soil, SSP and APP application did not increase the root diameter, but increased the specific root length (**Figures 4B, C**). Qiu et al. (2023) reported that salt stress decreased the root diameter in alfalfa. In the present study, alfalfa experienced more severe salt stress in the SSP and APP treatments than the other P treatments, which may be responsible for the root diameter change in saline–alkali soil.

### 4.3 Rhizosphere processes

Carboxylate exudation is an adaption strategy used by P-deficient plants to mobilize soil P (Lambers et al., 2006; Shen et al., 2011). The species of carboxylates exuded varies among plants, for example, malate and citrate in white lupin and trans-aconitate for maize (Li et al., 2010). Citrate is a more efficient carboxylate than others because it contains three carboxyl groups (Ryan et al., 2001). Similar to observations in white lupin and faba bean, in alfalfa, citrate was one of main carboxylates in the rhizosphere in sandy soil (**Table 1**). P fertilizers did not significantly affect the citrate exudation rate, which contradicted previous reports that P application promoted citrate exudation in faba bean but inhibited citrate exudation in white lupin (Li et al., 2008, Li et al., 2010). Salt stress has been reported to decrease the citrate content of *Puccinellia tenuiflora* (Shi et al., 2002), which is consistent with our results. However, the other carboxylates were not affected by salt stress. Consistent with the findings of Lipton et al. (1987), P-deficient alfalfa showed significantly enhanced succinate exudation in sandy soil. In saline–alkali soil, salt stress inhibited succinate exudation of alfalfa in the control treatment and led to similar concentrations in all P fertilizer treatments (**Table 1**).

Consistent with previous findings, in the present study, APase activity decreased with increasing shoot P concentration in alfalfa (Fraser et al., 2014; Richardson and Simpson, 2011). Higher APase activity was found in saline–alkali soil than sandy soil (**Figure 6A**), indicating more severe P-deficient stress for alfalfa grown in saline–alkali soil. SSP showed a weaker ability to reduce APase secretion than other fertilizers. However, P nutrition status cannot fully explain this finding. Several studies have shown that P-deficient plants tend to mobilize soil P through rhizosphere acidification (Hinsinger, 2001; Hinsinger et al., 2003; Li et al., 2010). It was confirmed by this study even alfalfa was supplied by nitrate in the control. NH_4_^+^ uptake by roots enhances rhizosphere acidification (Wang et al., 2022). However, APP but not DAP caused stronger rhizosphere acidification (**Figure 6B**), which was likely because NH_4_^+^ in DAP is easier to be nitrified than APP, because the latter is a slow-release fertilizer. It should be noted soil pH can be changed by fertilizer types applied in this study. SSP and KP are acidic fertilizers, which can bring protons into soil. DAP as an alkali fertilizer can increase soil pH. APP is a neutral fertilizer and cannot induce any change of soil pH. Moreover, NH_4_^+^ nitrification rate also affects soil pH during plant growth, especially rhizosphere pH, by changing NH_4_^+^/NO_3_^-^ ratio. The NO_3_^-^ uptake of roots generally causes increase of rhizosphere pH. In this study, NO_3_^-^ supply should weaken rhizosphere acidification in control, SSP and KP treatments which were supplied by Ca(NO_3_)_2_·4H_2_O as a compensated nitrogen.

## 5 Conclusions

Both soil type and P fertilizer species regulated the responses of root morphology and rhizosphere processes in alfalfa to soil P supply. P fertilizer application enhanced plant growth and P uptake to a greater extent in sandy soil than saline–alkali soil. Salt stress significantly inhibited P fertilizer efficiency in improving plant P nutrition. DAP and KP were observed to be the most efficient fertilizers in both sandy soil and saline–alkali soil. Considering the price of fertilizer, DAP can be the first choice in alfalfa production for farmers. Alfalfa plants applied with the other P fertilizers tended to modify root growth and rhizosphere processes to mobilize soil P and then increase plant P uptake. Thus, it is necessary to choose a suitable P fertilizer according to soil type for alfalfa production.

## Data availability statement

The raw data supporting the conclusions of this article will be made available by the authors, without undue reservation.

## Author contributions

TZ: Writing – original draft, Writing – review & editing, Data curation, Investigation, Software. WW: Conceptualization, Writing – review & editing. ZS: Supervision, Writing – review & editing. HL: Writing – review & editing, Conceptualization.
