# Supplementary material for: Phosphorus uptake and rhizosphere properties of alfalfa in response to phosphorus fertilizer types in sandy soil and saline-alkali soil
Source: Front Plant Sci. 2024 May 10;15:1377626. doi: 10.3389/fpls.2024.1377626 (PMC11122017; doi:10.3389/fpls.2024.1377626)
Supplement: Supplementary file 1 [file DataSheet_1.docx]

Supplementary Material

**
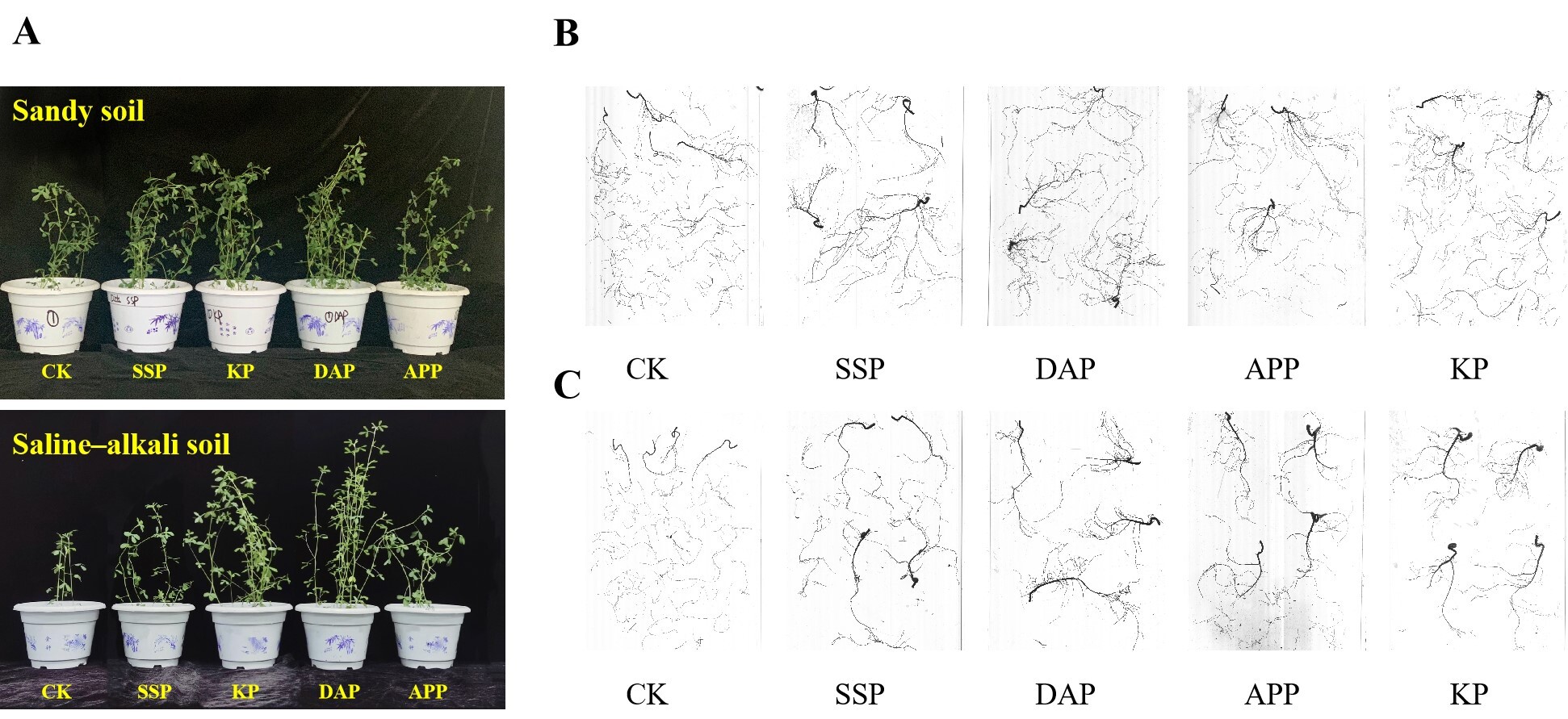
**

**FIGURE. S1** Photographs and scanned root images of alfalfa under different treatments. Plant photographs (**A**) and root images in sandy soil (**B**) and saline–alkali soil (**C**).
